# Characterization of the Spatial and Temporal Dispersion Differences Between Exhaled E-Cigarette Mist and Cigarette Smoke

**DOI:** 10.1093/ntr/nty121

**Published:** 2018-06-19

**Authors:** Dainius Martuzevicius, Tadas Prasauskas, Ari Setyan, Grant O’Connell, Xavier Cahours, Rémi Julien, Stéphane Colard

**Affiliations:** 1 Department of Environmental Technology, Kaunas University of Technology, Kaunas, Lithuania; 2 Empa (Swiss Federal Laboratories for Materials Science and Technology), Laboratory for Advanced Analytical Technologies, Dübendorf, Switzerland; 3 ETH Zürich, Institute of Environmental Engineering, Zürich, Switzerland; 4 Fontem Ventures B.V., Amsterdam, The Netherlands; 5 SEITA-Imperial Brands, Fleury-les-Aubrais, France

## Abstract

**Introduction:**

There are fundamental differences between electronic cigarettes (e-cigarettes) and conventional cigarette product categories with regards to potential environmental exposures, notably that e-cigarettes do not contain tobacco or generate side-stream emissions. Here we assess the spatial and temporal patterns of exhaled e-cigarette aerosol at a bystander’s position, and compare it with conventional cigarette smoke emissions.

**Methods:**

Smokers were asked to use e-cigarettes or smoke conventional cigarettes in a room-simulating chamber. Volunteers used the products at different distances from a heated mannequin, representing a bystander, and under different room ventilation rates. Aerosol particle concentrations and size distributions at the bystander’s position were measured.

**Results:**

For both product categories, the particle concentrations registered following each puff were in the same order of magnitude. However, for e-cigarettes the particle concentration returned rapidly to background values within seconds; for conventional cigarettes it increased with successive puffs, returning to background levels after 30–45 minutes. Unlike for the e-cigarette devices tested, such temporal variation was dependent on the room ventilation rate. Particle size measurements showed that exhaled e-cigarette particles were smaller than those emitted during smoking conventional cigarettes and evaporated almost immediately after exhalation, thus affecting the removal of particles through evaporation rather than displacement by ventilation.

**Conclusions:**

Significant differences between emissions from the tested e- and conventional cigarettes are reported. Exhaled e-cigarette particles are liquid droplets evaporating rapidly; conventional cigarette smoke particles are far more stable and linger.

**Implications:**

• Several factors potentially influencing particle behavior after exhalation of e-cigarette aerosols or emitted during smoking conventional cigarettes were studied.

• Differences in particle size between those exhaled following use of e-cigarettes and those emitted during smoking of conventional cigarettes were observed.

• E-cigarette particle concentrations decreased rapidly following exhalation due to evaporation.

• The removal of particles following smoking conventional cigarettes was much slower and was dependent on the room ventilation rate.

## Introduction

Electronic cigarettes (e-cigarettes) have been characterized by Public Health England as being around 95% less harmful than conventional cigarettes^1^ with recent research showing that these devices can assist smokers in replacing conventional cigarettes and reducing their cigarette per day consumption.^2,3^

E-cigarettes are battery-powered devices that have cartridges prefilled by manufacturers or refillable tanks containing a liquid mixture composed primarily of propylene glycol and/or glycerol, nicotine, and flavorings.^4^ During use, inhalation activates a pressure-sensitive circuit that heats the atomizer and turns the liquid into an aerosol (popularly referred to as “vapor”). The aerosol is then inhaled by the user through the mouthpiece and exhaled as a fine mist. Some e-cigarette devices have a button that enables the user to manually activate the heating element.

Because e-cigarettes do not burn (or contain) tobacco, no side-stream emissions or any tobacco smoke is produced. Only what is exhaled by e-cigarettes users enters the surrounding air. With the increasing prevalence of e-cigarettes among smokers worldwide, there is growing discussion among public health organizations and the scientific community as to whether the aerosol exhaled following use of such products has implications for the quality of air breathed by bystanders through so-called “passive vaping.”^5^ A number of studies have shown that compared with conventional tobacco cigarettes, exhaled e-cigarettes aerosols release very low levels of chemicals into the ambient air^6–9^ and are unlikely to pose an issue to bystanders based on regulatory indoor air quality standards.^1,10,11^

Exhaled e-cigarette aerosols have also been reported to consist of fine and ultrafine “particles” of particulate matter^12,13^; however, the term “particle” does not distinguish between solid particles (eg, those emitted from fuel combustion in engines or furnaces) and liquid droplets. Exhaled e-cigarette “particles” have been shown to be liquid droplets primarily composed of propylene glycol, glycerol, and water which condense into a visible mist on exhalation.^12,14,15^ In a small 8 m^3^ experimental chamber, the rapidly changing nature of exhaled e-cigarette aerosol droplets has been observed.^6^ Moreover, following release of e-cigarette aerosols into a larger experimental chamber, droplets in the range 20–300 nm have been recorded, reaching a peak concentration from 10^3^ to 10^6^ particles/cm^3^,^6,16^ depending on the generation of tested device, temporal resolution of measurement, ventilation pattern, and other parameters. The short half-life of e-cigarette aerosols in ambient air has been reported to be around 10 seconds (ie, >100 times faster than conventional cigarette smoke) due to the rapid evaporation of liquid droplets at room temperature.^12,17^

At this time, there is limited data available on the dynamic properties of exhaled e-cigarette aerosols, in particular the exhaled “particles,” and how they differ from those released during the smoking of a conventional cigarette (which continuously emits particles from the combustion process at the lit end of a cigarette itself). To that end, we aimed to investigate the spatial and temporal variations of “particles” exhaled following the use of commercially available e-cigarettes and those released during the smoking of conventional cigarettes in a chamber under controlled environmental conditions.

## Methods

### Experimental Room

**Figure 1.**
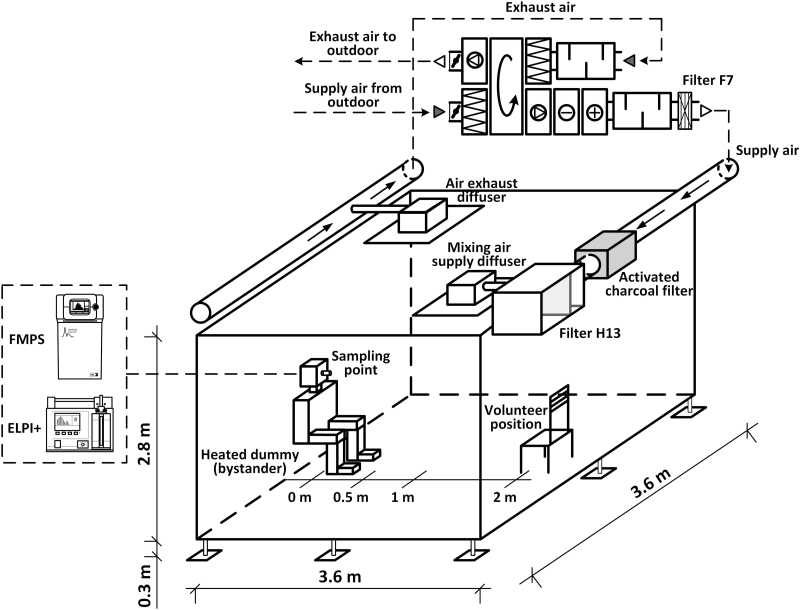
Room-simulating chamber with heated mannequin to simulate a seated bystander under controlled ventilation.

A walk-in room-simulating environmental chamber was used in this study (surface area, 13 m^2^; volume, 35.8 m^3^) (Figure 1). The walls, floor, and ceiling of the chamber were fabricated using conventional construction materials, such as painted drywall, polyvinyl chloride lining, and a panel ceiling. The chamber allowed precise control of temperature, relative humidity, and air exchange rate. The chamber temperature was 19°C–23°C and the relative humidity 30%–38%. The chamber was equipped with in-ceiling air exhaust diffuser as well as air supply system, consisting of in-ceiling diffuser. Supply and exhaust airflows, as well as supply air temperature, were controlled using the air handling unit (GOLD 04, Swegon AB, Sweden). The supply air temperature during the experiments was set to around +20°C.

A heated mannequin of rectangular geometry was installed in the chamber to simulate the position of a seated bystander with the inclusion of “legs” considering that this was previously documented as an important factor having influence on airflows around a person.^18^ The surface area of the mannequin was equal to 1.7 m^2^; it was covered with a textile fabric and the surface temperature of the mannequin was in the range of +31°C to +34°C, similar to the human body surface temperature.^19^ The mannequin was seated on a wooden chair.

The air samples were drawn through a grounded copper inlet tube mounted at the bystander’s mouth position (corresponding to the breathing zone) and divided to aerosol instruments, which were positioned immediately outside of the chamber to minimize particle losses due to diffusion and evaporation processes. The ventilation air supplied to the chamber was conditioned in a heat exchanger and treated with three steps of filtration—prefilter of class F7, activated carbon filter, and HEPA H13 final filter. Four-way mixing ventilation was chosen for this study as it is commonly used in residential and office buildings. Three ventilation rates of 0, 1, and 2 air changes per hour were tested. One multinozzle air supply diffuser of 0.5 × 0.5 m with plenum box was used for the in-ceiling air supply case. The four-way mixing was created by directing the nozzles in four directions. Air distribution patterns in chamber were tested before the experiments using artificial aerosol generated by a fog machine using a water- and glycol-based fluid.

Before the start of each run, a background control particle number concentration (PNC) was recorded (3 minutes before the start). Low concentration of aerosol particles (<300 particles/cm^3^) and gaseous organic ambient chemicals (as total volatile organic compounds) in the supply air was ensured. After the end of each experiment, the room was purged with fresh air until the concentrations of aerosol particles reached the background values (ie, the observed values before the initiation of the experiment). The effects of aerosol dispersion were monitored for sufficient time to reach the lower asymptote concentration of the exponential decay.

### Measurement Methods

The real-time size-segregated PNC and samples were drawn using the fast mobility particle sizer (FMPS) Model 3091 spectrometer (TSI, Inc, Shoreview, MN) and the electrical low pressure impactor (Dekati Inc, Kangasala, Finland). Both instruments operated with the time resolution of 1 second. The FMPS measured aerosol particles in the range from 5.6 to 560 nm, offering a total of 32 channels of resolution based on the electrical mobility measurements. The particle size was calibrated with polystyrene latex (PSL) solutions in the range 50–300 nm, and size distributions measured by the FMPS were postcorrected using the relationship between the particle sizes measured by the instrument and the real sizes of the PSL samples. The FMPS operated at a sample flow rate of 10 L/min, greatly reducing particle sampling losses due to diffusion, with time resolution of one size distribution per second. Electrical low pressure impactor operates at a high sample flow rate of 10 L/min. Electrical low pressure impactor divided aerosol particles to 15 fractions (from 0.006 to 10.0 µm), based on the aerodynamic diameter. Aerosol samples were collected on greased 25 mm diameter aluminum foil substrates.

### Products

All e-cigarettes were commercially available (manufactured by Fontem Ventures, the Netherlands) and purchased from a number of UK retail outlets at the time of the study. E-cigarette 1 was the Puritane 1.6% nicotine tobacco flavored closed system disposable e-cigarette (termed “cig-a-like disposable”); e-cigarette 2 was the blu PLUS+ 1.8% nicotine tobacco flavored closed system rechargeable e-cigarette (termed “cig-a-like cartomizer”); and e-cigarette 3 was the blu PRO refillable rechargeable open system e-cigarette containing 1.8% nicotine tobacco flavored e-liquid.

For the conventional cigarette comparator, the market leading brand Marlboro Gold (Philip Morris International, Inc) was selected with reported tar, nicotine, and carbon monoxide yields (as obtained under ISO3308 smoking regime) yields of 8 mg, 0.6 mg, and 9 mg, respectively.

## Volunteers

Three smokers of conventional cigarettes (age: 30–45 years, all male) who were also regular experienced users of e-cigarettes participated in this study. The volunteers were informed about the course of the study and the voluntary nature of their participation in both written and verbal form. The volunteers gave their written informed consent for their participation prior to the study commencing.

### Experiment Plan and Quality Assurance

The experiment was performed as a randomized full-factorial design, supplemented with additional runs aiming to compare or validate main experimental runs (Supplementary Table 1).

The ventilation rate and positioning of the experimental equipment were adjusted to appropriate settings. Air velocity and temperature temporal gradients were measured at each setup of ventilation. The majority of experiments was conducted under the four-way mixing ventilation supply. Several runs were repeated using the full mixing in the chamber using additional fans to simulate ideal mixing conditions with the aim to test the effect of the positioning of emissions source and sampling inlet.

The volunteers entered the chamber and smoked the conventional cigarette or vaped the e-cigarettes at different distances from the mannequin. To facilitate the comparison between volunteers as well as between the e-cigarettes and conventional cigarette, the number of puffs and the interval between puffs were defined: for conventional cigarette—one puff every 30 seconds, total number of five puffs, and extinguish the cigarette in an ashtray; for e-cigarette—one puff every 30 seconds, total number of five puffs. No instructions on puff duration, puff volume, or inhalation on mouth-hold were issued. As topography (consumption behavior) studies have shown there are significant differences in user puffing behaviors associated with conventional cigarette and e-cigarette use—that is, e-cigarettes are not used in the same way as conventional cigarettes are smoked^20^—no instruction was given on puff and exhalation durations or aerosol inhalation per mouth-hold for any product type. This allows for the results to include typical user puffing behavior variations.

The real-time measurements of PNC were compared using the FMPS and Electrical low pressure impactor; the obtained particle size distributions were comparable and consistent among the tested runs. Moreover, selected runs were repeated to estimate the repeatability of the measurements, with relative error of the repeated measurements less than 20%.

The obtained PNCs were used to estimate PNC decay rates. These generally represent interaction between ventilation, gravitational settling, diffusion, electrostatic effects, and thermophoresis. Among these, the removal of aerosol particles due to ventilation and surface deposition is the most important. Temporal variation of particle concentrations was described by the solution of the first order differential mass balance equation, namely $C_{t}=C_{0}e^{-kt}$, where *k* is the particle concentration decay rate (per minute), *t* is the time (minutes), *C*_0_ is the initial particle concentration, and *C*_*t*_ is particle concentration (particles per cubic centimeter) measured at the time *t*.

## Results

### Temporal Variations of PNC

**Figure 2.**
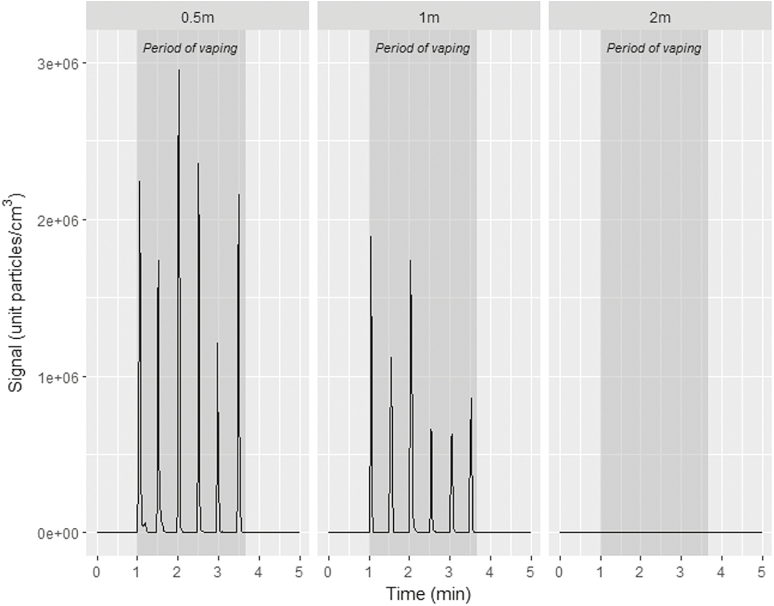
Temporal variation of particle number concentration at the bystander’s position during vaping of cig-a-like disposable e-cigarette (e-cigarette 1) at different distances between the vaper and the bystander (0.5, 1, and 2 m). Air changes per hour = 1.

Figure 2 shows the temporal variation of PNC at the bystander’s position during use of e-cigarette 1 at different distances between the vaper and the bystander (0.5, 1, and 2 m) with one air changes per hour. The data obtained from one volunteer with use of e-cigarette 1 is presented; however, the patterns are representative for the other volunteers using this e-cigarette and for use of the other e-cigarette devices tested.

In general, e-cigarette puffing periods can be distinguished into two clear “puff phases” for both closed and open e-cigarette devices systems: (1) a rapid increase in PNC by several orders of magnitude over background levels, up to 10^6^ particles/cm^3^ followed by (2) a very rapid decrease to almost to a background level within seconds (consistent with the findings from Zhao et al.^21^).

Such pattern of temporal variation was evident at a distance of 0.5 and 1 m from the bystander, where the bystander experienced a direct exhaled puff into the breathing zone. The duration of the increase–decrease episode was in range of 4–8 seconds, which corresponded to the duration of exhalation of the puff plus the time taken for the aerosol to reach the sampler. At a distance of 2 m, the impact of exhaled puffs was not clearly observable. This is likely due to the fact that the aerosol particles were not able to reach the bystander’s position once exhaled due to the dispersion in the volume of the room together with rapid evaporation.

**Figure 3.**
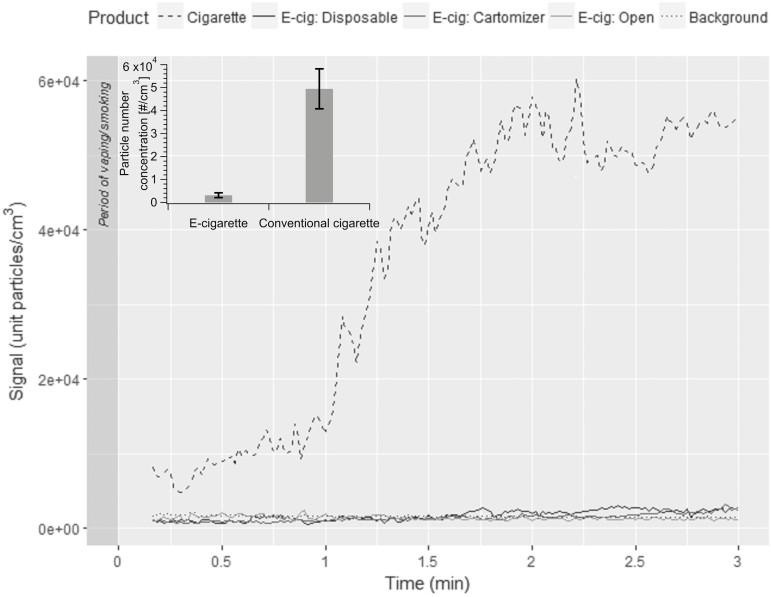
Temporal variation of particle number concentration at the bystander position starting at 20 seconds after smoking a conventional cigarette or vaping different e-cigarette types. Distance between the vaper and the bystander = 0.5 m. Air changes per hour = 1.

The immediate decrease in PNC after a single puff may be explained by several factors: (1) the dispersion of aerosol particles within the chamber due to thermal fields of the bystander mannequin plus chamber ventilation, (2) the deposition of exhaled aerosol particles on to surrounding surfaces, or (3) evaporation of exhaled aerosol particles to vapor phase compounds. The dispersion mechanism only partially explains such rapid variations in PNC levels. Even if particles were dispersed in the room volume, this would be expected to result in a clear increase in background concentrations. The latter phenomenon has been observed in case of conventional cigarettes: After puffing was over, the PNC decreased, but was then followed by an increase and subsequent decrease. This phenomenon is attributed to the dispersion of smoke particles within the room. Due to a nonvolatile and stable nature of tobacco smoke particles (in the exhaled cigarette smoke + side-stream emissions), they are likely to reside in the room until removed by ventilation or potential deposition on surfaces. By contrast, for all e-cigarettes the PNC after five puffs remained nearly at the same value as before the experiment. This suggests there was no residual particle concentration in the chamber. Figure 3 shows the PNC at the bystander’s position starting at 20 seconds after smoking or vaping the different product types with a distance between the vaper and the bystander of 0.5 m and air changes per hour of 1.

For all e-cigarette types studied, the PNC always returned to background levels (<1000 particles/cm^3^) within ~10–15 seconds after the vaping session and was independent of the chamber ventilation rate. By contrast, with conventional cigarettes, the PNC returned to background levels after 30–45 minutes and was dependent on the exposure chamber ventilation rate.

### Decay Rates of PNC

Quantitative assessment of PNC decay following a single puff confirmed a very rapid decrease in concentration at a rate of 15–30/min, or 900–1800/h. Such high values for the decay rates were applicable to all e-cigarette devices and conventional cigarette at a close distance to a bystander. A distance of 2 m resulted in lower decay rates due to a longer travelling time of aerosol particles (liquid or solid) to the bystander’s position and dispersion within the chamber.

An interesting observation in the decay rate after puffing is also noted. In the case for all e-cigarettes tested, the decay varied in a range of 0.1–0.2/min (which was about 150 times lower compared to the initial decay), or could not be calculated because no measurable decay was observed. However, in case of conventional cigarettes, after puffing the decay was prominent in all locations and resulted in values less than 0.1/min (400 times lower compared to the initial decay), indicating substantially slower removal of aerosol particles from the chamber.

The PNC decay rates were analyzed with respect to the influence of controlled factors, namely volunteers, ventilation rates, and distances from the bystander. In case of all e-cigarettes tested, the distance from the bystander and (to a lower extent) different volunteer puffing topographies played the major roles in determining the variation of decay rates (*p* < .05, Kruskal–Wallis analysis of variance), while ventilation rate was not a significant factor (*p* > .05). At a distance of 2 m, significantly slower decay rates were observed due to smaller maximum concentration (at the levels of 10^4^–10^5^ particles/cm^3^) reached at the bystander and the dispersion of particles in the volume of the room while they travel to the bystander’s position.

### Particle Size Distributions

**Figure 4.**
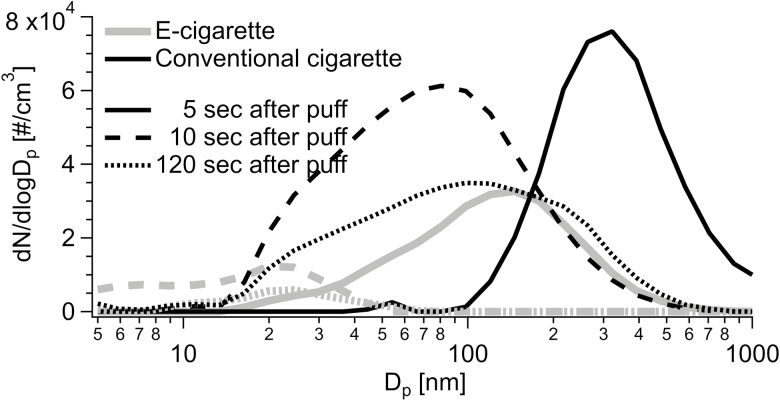
Temporal variation of particle size distribution at the bystander’s position after exhalation of cig-a-like disposable e-cigarette puff. 0 second corresponds to the maximum of particle number concentration at the bystander position. Distance between the vaper and the bystander = 0.5 m. Air changes per hour = 1.

We observed some differences between the conventional cigarette and e-cigarette products tested in terms of particle size distribution (Figure 4). At a short distance between the volunteer and the bystander, particles emitted during smoking a conventional cigarette usually had a mode at 300 nm, whereas those exhaled following use of the different e-cigarette products had a smaller mode, around 150 nm, as well as having a second mode at the lowest channels (20–30 nm). Recently two modes for exhaled e-cigarette droplets at 15 and 85 nm were reported.^21^ Following e-cigarette aerosol exhalations, the larger mode shifted quickly to smaller sizes. Figure 4 shows the temporal variation of particle size distribution at the bystander’s position after exhalation of an e-cigarette puff at the shortest distance. In Figure 4, 0 second corresponds to the maximum of PNC at the bystander’s position. The observed rapid decrease in particle size is due to the fact that the exhaled e-cigarette (representative for all e-cigarette devices studied) particles were liquid droplets evaporating rapidly with the time.

The ventilation rate within the exposure chamber did not influence significantly the particle size distribution for both e-cigarette and conventional cigarette. Generally, a higher supply rate of ventilation air would be expected to increase the rate of particle evaporation due to the more effective removal of gas-phase volatile organic compounds and thus providing lower partial pressure of these compounds in gas phase. However, the rate of the introduction of fresh air is much slower compared to the very fast processes of particle evaporation, and thus the effect of ventilation was not significant.

## Discussion

Experiments performed in the exposure chamber provide a greater understanding of the dynamic properties of “particles” emitted during smoking conventional cigarettes and exhaled following use of e-cigarettes, and show the different spatial and temporal profiles for each product type. The influence of several parameters was tested during this study: the product types, volunteers, the ventilation rate, and the distance between the volunteer and the bystander. The variation of aerosol particle concentrations was very rapid and was associated with the direct impact of puffs for all products studied, including conventional cigarette. The concentration increased by several orders of magnitude from the background and dropped back to background concentration within 10 seconds during use of the e-cigarette products studied with no further increase in concentration. For the conventional cigarette, a similar profile was registered but was followed by an increase in overall background concentration in a chamber reflecting the stable nature of conventional cigarette combustion particles.

The concentration of particles in the air released after each puff was in the same order of magnitude for all the product types (10^6^ particles/cm^3^). However, after successive puffs, the particle concentration increased up to 50 000 particles/cm^3^ with the conventional cigarette, while it stayed at background levels (< 1000 particles/cm^3^) with the e-cigarettes tested. We also observed significant differences between conventional and e-cigarettes in terms of particle size. Indeed, with the conventional cigarette, released particles had a main mode at around 300 nm, and this particle size distribution was not affected by the distance between the volunteer and the bystander. Particles exhaled after the use of all e-cigarettes tested were smaller (around 150 nm), and a shrink of the particle size at a greater distance from the bystander was observed.

Consistent with the findings from Bertholon et al.^17^ and Zhao et al.,^21^ our results confirm that the “particles” exhaled during use of e-cigarettes are highly volatile droplets that evaporate within several seconds following exhalation unlike for conventional cigarettes. Such results were obtained using human volunteers to generate true exhaled aerosol, rather than machine generated, thus creating realistic use conditions. Within 2 m from an e-cigarette user, the probability of inhalation of particle-phase compounds decreases significantly, as compared to 1 m or closer, indicating a potential minimum exposure associated with particles as air pollutants. The variations of particles in the indoor air resulting from vaping may only be registered by direct reading aerosol instrumentation, having high temporal resolution (not less than 1 second) and capable in measuring particles smaller than 300 nm in diameter. Conversely, filter-based reference methods that take into account longer sampling durations, conditioning of filters, and gravimetric determination of particles will probably be unable to detect significant changes of filter mass (proportional to particle concentration) purely due to evaporation.

The methodology developed in this article may be further applied to the researching particle emissions in other types of e-cigarette products, because the composition of vaping liquid as well as the technology used for the generation of aerosols may influence the spatial and temporal profiles of exhaled aerosols. Some differences may also be associated with different e-cigarette user puffing topographies and the number of users in a given room, where the effects of particle coagulation may occur at higher concentrations of exhaled aerosol. Moreover, the effects of ambient temperature and relative humidity may also be considered as physical parameters affecting vapor pressure of volatile compounds in the exhaled liquid droplets.

It should also be noted that the concentration of volatile organic compounds has been previously observed to increase in indoor air with the evaporation of particle-phase compounds. An increased gas-phase nicotine, glycerol, and propylene glycol,^9,10^ and trace concentrations of aldehydes (as by-products of the thermal breakdown of e-liquid ingredients^16^) have been reported in the ambient air following e-cigarette use albeit well below indoor air quality guidelines. These observations are not expected to be associated with particle-specific effects.

## Conclusions

Our results suggest that particles exhaled following use of the e-cigarette devices tested are actually liquid droplets constituted of volatile compounds from the e-liquid. These particles evaporate very fast and disappear 10–15 seconds after the puff, transferring to vapor volatile organic compounds. By contrast, particles from conventional cigarettes are mainly non- or semi-volatile particles that are much more stable than those from e-cigarettes. The removal of these particles is much longer (minutes) and is dependent on the ventilation rate in the room.

The data presented here highlight several factors influencing particle dynamics following exhalation of e-cigarette aerosol and show the clear and substantial differences between e-cigarettes and conventional cigarettes. This study was conducted in a controlled environment with constant environmental conditions (temperature and relative humidity) and should be validated in real-world environments of changing environmental parameters to better understand the phenomenon of particle dynamics.

The results presented here relate to typical closed and open (tank) system devices and may not be generally applicable to other products such as advanced personal vaporizers (MODs), user topographies, and technology difference may also impact exhaled aerosol characteristics/properties. Further research on different product types is warranted in different settings with different volunteer groups.

## Supplementary Material

Supplementary data are available at Nicotine & Tobacco Research online.

## Funding

The work in this article was supported by Fontem Ventures B.V. Imperial Brands Group plc is the parent company of Fontem Ventures B.V., the manufacturer of the e-cigarette products used in this study. The work presented in this article was performed in Lithuania in accordance with the Code of Academic Ethics of the Kaunas University of Technology, Lithuania.

## Declaration of Interests

*GC, XC, RJ, and SC are full time employees of Imperial Brands Group plc*.

## Supplementary Material

nty121_suppl_Supplementary_Material
