# Supplementary material for: Characterization of the Spatial and Temporal Dispersion Differences Between Exhaled E-Cigarette Mist and Cigarette Smoke
Source: Nicotine Tob Res. 2018 Jun 19;21(10):1371–7. doi: 10.1093/ntr/nty121 (PMC6751519; doi:10.1093/ntr/nty121)
Supplement: nty121_suppl_Supplementary_Material [file nty121_suppl_supplementary_material.docx]

**Characterisation of the spatial and temporal dispersion differences between exhaled e-cigarette mist and cigarette smoke**

Dainius Martuzevicius, Tadas Prasauskas, Ari Setyan, Grant O’Connell, Xavier Cahours, Rémi Julien, Stéphane Colard

**SUPPLEMENTARY MATERIAL**

Table S1. Experimental plan and main results on particle size, concentration and temporal variation during the usage of nicotine products in the chamber

| **Exp No** | **Cigarette type** | **Volunteer** | **Ventilation intensity, ach** | **Distance to bystander, m** | **Mode of Particle Size, nm** | **Maximum Concentration, particles/cm3** | **Concentration Decay Rate 1/min** | **Concentration Growth Rate 1/min** |
| --- | --- | --- | --- | --- | --- | --- | --- | --- |
| 1 | Conventional | v1 | 1 | 0.5 | 165 | 5.98E+07 | 34.6 | 140 |
| 2 | Conventional | v1 | 1 | 2 | 165 | 8.29E+05 | 20.6 | 83 |
| 3 | Conventional | v1 | 2 | 0.5 | 165 | 2.43E+08 | 41.9 | 169 |
| 4 | Conventional | v1 | 2 | 2 | 165 | 5.85E+05 | 4.8 | 19 |
| 5 | Conventional | v2 | 1 | 0.5 | 165 | 2.61E+08 | 44 | 178 |
| 6 | Conventional | v2 | 1 | 2 | 165 | 7.35E+05 | 9 | 36 |
| 7 | Conventional | v2 | 2 | 0.5 | 165 | 1.35E+08 | 37.2 | 150 |
| 8 | Conventional | v2 | 2 | 2 | 165 | 2.44E+06 | 19.4 | 78 |
| 9 | Conventional | v3 | 1 | 0.5 | 165 | 1.34E+08 | 39.6 | 160 |
| 10 | Conventional | v3 | 1 | 2 | 165 | 8.07E+05 | 8.4 | 33 |
| 11 | Conventional | v3 | 2 | 0.5 | 165 | 4.73E+07 | 36.9 | 149 |
| 12 | Conventional | v3 | 2 | 2 | 165 | 5.61E+05 | 1.5 | 6 |
| 13 | Electronic | v1 | 1 | 0.5 | 165 | 1.01E+07 | 23.8 | 92.3 |
| 14 | Electronic | v1 | 1 | 1 | 165 | 3.59E+07 | 28.2 | 121.2 |
| 15 | Electronic | v1 | 1 | 2 | 165 | 2.90E+05 | 9.3 | 36.1 |
| 16 | Electronic | v1 | 2 | 0.5 | 380 | 4.41E+06 | 19.7 | 98.6 |
| 17 | Electronic | v1 | 2 | 1 | 165 | 2.93E+06 | 29.4 | 113.1 |
| 18 | Electronic | v1 | 2 | 2 | 165 | 7.41E+04 | 6.1 | 10 |
| 19 | Electronic | v2 | 1 | 0.5 | 165 | 7.38E+06 | 26.3 | 101.8 |
| 20 | Electronic | v2 | 1 | 1 | 165 | 6.72E+06 | 29 | 129.3 |
| 21 | Electronic | v2 | 1 | 2 | 165 | 2.31E+04 | 1.5 | 4.7 |
| 22 | Electronic | v2 | 2 | 0.5 | 165 | 6.64E+06 | 31.5 | 121.2 |
| 23 | Electronic | v2 | 2 | 1 | 165 | 8.47E+06 | 31.1 | 84.9 |
| 24 | Electronic | v2 | 2 | 2 | 165 | 3.09E+04 | 4.6 | 11.2 |
| 25 | Electronic | v3 | 1 | 0.5 | 165 | 1.27E+06 | 18.4 | 77.6 |
| 26 | Electronic | v3 | 1 | 1 | 165 | 2.23E+04 | 3.2 | 12.8 |
| 27 | Electronic | v3 | 1 | 2 | 165 | 2.44E+04 | 4.7 | 5.2 |
| 28 | Electronic | v3 | 2 | 0.5 | 380 | 2.53E+06 | 14.9 | 51 |
| 29 | Electronic | v3 | 2 | 1 | 165 | 2.01E+04 | 1.7 | 1.8 |
| 30 | Electronic | v3 | 2 | 2 | 165 | 3.17E+04 | 3.1 | 6.6 |
